# Superoxide dismutase activity is significantly lower in end-stage osteoarthritic cartilage than non-osteoarthritic cartilage

**DOI:** 10.1371/journal.pone.0203944

**Published:** 2018-09-17

**Authors:** Masato Koike, Hidetoshi Nojiri, Hiroaki Kanazawa, Hiroto Yamaguchi, Kei Miyagawa, Nana Nagura, Sammy Banno, Yoshiyuki Iwase, Hisashi Kurosawa, Kazuo Kaneko

**Affiliations:** 1 Department of Orthopaedic Surgery, Juntendo Tokyo Koto Geriatric Medical Center, Tokyo, Japan; 2 Department of Orthopaedic Surgery, Juntendo University Graduate School of Medicine, Tokyo, Japan; University of Umeå, SWEDEN

## Abstract

Recent studies have shown that superoxide dismutase 1 (SOD1), SOD2, and SOD3 are significantly decreased in human osteoarthritic cartilage. SOD activity is a marker that can be used to comprehensively evaluate the enzymatic capacities of SOD1, SOD2, and SOD3; however, the trend of SOD activity in end-stage osteoarthritic tissues remains unknown. In the present study, we found that SOD activity in end-stage osteoarthritic synovium of the knee was significantly lower than that in control synovium without the influence of age. The SOD activity was significantly lower in the end-stage knee osteoarthritic cartilage than in the control, but a weak negative correlation was observed between aging and SOD activity. However, SOD activity in end-stage hip osteoarthritic cartilage was significantly lower than that in control cartilage without the influence of aging. The relationship between osteoarthritis and SOD activity was stronger than the relationship between aging and SOD activity. These results indicate that direct regulation of SOD activity in joint tissues may lead to suppression of osteoarthritis progression.

## Introduction

Osteoarthritis (OA) is a chronic degenerative disease characterized by cartilage degeneration. Many people worldwide experience OA-related pain [1]. Thus, biomarkers with which to evaluate OA activity, OA prognosis, OA progression, and treatment responses are required [2, 3].

In recent years, oxidative stress has attracted attention as a cause of OA [4, 5]. Various enzymatic antioxidants exist in the intracellular and extracellular environments. One such enzymatic antioxidant, superoxide dismutase (SOD), eliminates the reactive oxygen species (ROS) superoxide and has received particular attention in the treatment of OA [6]. SOD is an enzymatic antioxidant involved in the conversion of superoxide anion radical (O_2_^−^) into hydrogen peroxide and molecular oxygen, which is important for controlling the ROS level in cells. Three isozymes of SOD are known: SOD1, which is localized in the cytoplasm; SOD2, which is localized in the mitochondria; and SOD3, which is localized in the extracellular components. In 2009, Scott *et al*. reported that the protein and expression levels of SOD1, SOD2, and SOD3 are significantly lower in OA-affected cartilage than intact cartilage of the knee [6]. SOD2 has attracted particular attention; some researchers have reported that the SOD2 level is significantly decreased in OA-affected knee cartilage [6–8]. However, whether this decreased SOD2 level causes OA remains unclear. We previously concluded in an animal experiment involving chondrocyte-specific *Sod2*-knockout mice that a reduction in the level of the antioxidant enzyme SOD2, localized in the mitochondria, is a cause of cartilage degeneration [9].

SOD down-regulation in cartilage is important for the development of OA progression. However, whether SOD is a useful biomarker for OA is unknown. The enzymatic activity of an antioxidant is the most important parameter with which to determine its biological effects. In the present study, we focused on SOD activity, which can be used to comprehensively evaluate the enzymatic capacities of SOD1, SOD2, and SOD3, and investigated the trend of SOD activity in joint tissues including the knee articular cartilage, synovium, and femoral head cartilage. The purpose of this study was to examine the possibility of using SOD as an OA biomarker by examining the SOD activity in OA-affected knee cartilage and synovium from patients with end-stage knee OA and in OA-affected femoral head cartilage from patients with end-stage hip OA.

## Materials and methods

### Approval and informed consent

This study protocol was approved by the ethics committee of Juntendo Tokyo Koto Geriatric Medical Center. All experiments were conducted in accordance with the Declaration of Helsinki. Informed consent was obtained from all patients prior to the study.

### Quantification of dihydroethidium (DHE) staining

Cartilage and synovium specimens were taken from the outside of the patellofemoral joint with biopsy forceps (S1 Fig and S1 Table). Within 2 hours after completion of surgery, they were embedded in OCT compound and sectioned at a thickness of 10 μm. After drying sections at room temperature for several minutes, DHE (Life Technologies Corporation, Gaithersburg, MD, USA) staining was carried out simultaneously for control group and OA group specimens, and the results were then evaluated. DHE solution was prepared in phosphate-buffered saline (PBS). The obtained cartilage and synovium were stained for 30 min at 37°C under 20% O_2_ and 5% CO_2_ with 10 μM DHE solution to detect intracellular superoxide [9]. The stained cartilage and synovium were then washed with PBS, and the intracellular ROS level was detected using a BX51 microscope (Olympus, Tokyo, Japan) equipped with a 60×/1.0 NA objective (Nikon, Tokyo, Japan) and the NIS-Elements F 3.0 software package (Nikon) (camera settings: manual exposure, gain of 6.8 times per second). To eliminate the effects of extracellular staining, 100 cells were arbitrarily chosen and the average value of the fluorescence intensity was quantified using ImageJ (National Institutes of Health, Bethesda, MD, USA).

### Evaluation of SOD activity in knee articular cartilage and synovium

Cartilage and synovium obtained from the knee joint of patients who underwent total knee arthroplasty for end-stage knee OA were defined as end-stage knee OA cartilage and synovium. Cartilage and synovium obtained from the knee joint of patients who underwent knee arthroscopy for meniscal tears or anterior cruciate ligament reconstruction were defined as control specimens. The degree of OA was evaluated using the Kellgren–Lawrence classification [10]. Samples of cartilage and synovium were taken from the outside of the patellofemoral joint with biopsy forceps (S1 Fig and S2 Table). The exclusion criteria for both groups included a history of rheumatoid arthritis, local joint infection, or gout. Obtained cartilage specimens were promptly preserved at −80°C after surgery. The sample was thereafter melted at room temperature, and 300 μL of a physiological phosphate-buffered solution and two stainless steel balls were placed in a bead beater-type homogenizer (Beads Crusher μT-01; TAITEC Co., Ltd., Saitama, Japan) and the sample was crushed under 4,600 rpm × 30 s × two times. The homogenate was collected and centrifuged for 10 min at 4°C, 10,000 × *g*. Finally, the supernatant was collected and the SOD activity in the samples was analyzed using the NWLSS Superoxide Dismutase Activity Assay (Northwest Life Science Specialties, LLC, Vancouver, WA, USA) according to the manufacturer’s instructions. The results are expressed in units per mg protein. The protein amount was measured by the Protein Assay Rapid Kit WAKO (Wako Pure Chemical Industries, Ltd., Osaka, Japan) according to the manufacturer’s instructions.

### Evaluation of SOD activity and malondialdehyde (MDA) in hip articular cartilage

Cartilage taken from the femoral head of patients who underwent bipolar hip arthroplasty for femoral head neck fractures was defined as non-OA cartilage. Cartilage taken from the femoral head of patients who underwent total hip arthroplasty for hip OA was defined as hip OA cartilage. The exclusion criteria for both groups included a history of rheumatoid arthritis, local joint infection, or gout. Cartilage specimens were taken from the femoral head with biopsy forceps (S2 Fig and S3 Table). The obtained specimens were promptly preserved at −80°C after surgery. The sample was thereafter melted at room temperature, and 300 μL of a physiological phosphate-buffered solution and two stainless steel balls were placed in a bead beater-type homogenizer (Beads Crusher μT-01; TAITEC Co., Ltd.), and the sample was crushed under 4,600 rpm × 30 s × two times. The homogenate was then collected and centrifuged for 10 min at 4°C, 10,000 × *g*. Finally, the supernatant was collected, and the SOD activity in the samples was evaluated using a Superoxide Dismutase Assay Kit (Cayman Chemicals, Ann Arbor, MI, USA) according to the manufacturer’s instructions. The results are expressed in units per mg protein. The protein amount was measured by the Protein Assay Rapid Kit WAKO according to the manufacturer’s instructions. Additionally, the MDA level in the samples was evaluated using an MDA Assay Kit (Northwest Life Science Specialties, LLC) according to the manufacturer’s instructions.

### Statistical analysis

All statistical analyses were performed using SPSS Statistics 23 (IBM Corp., Armonk, NY, USA). Data are expressed as mean ± standard deviation. Statistical significance was defined as *P* < 0.05.

## Results

### Significantly higher intracellular ROS generation in knee cartilage of patients with OA than in control cartilage

**Fig 1:**
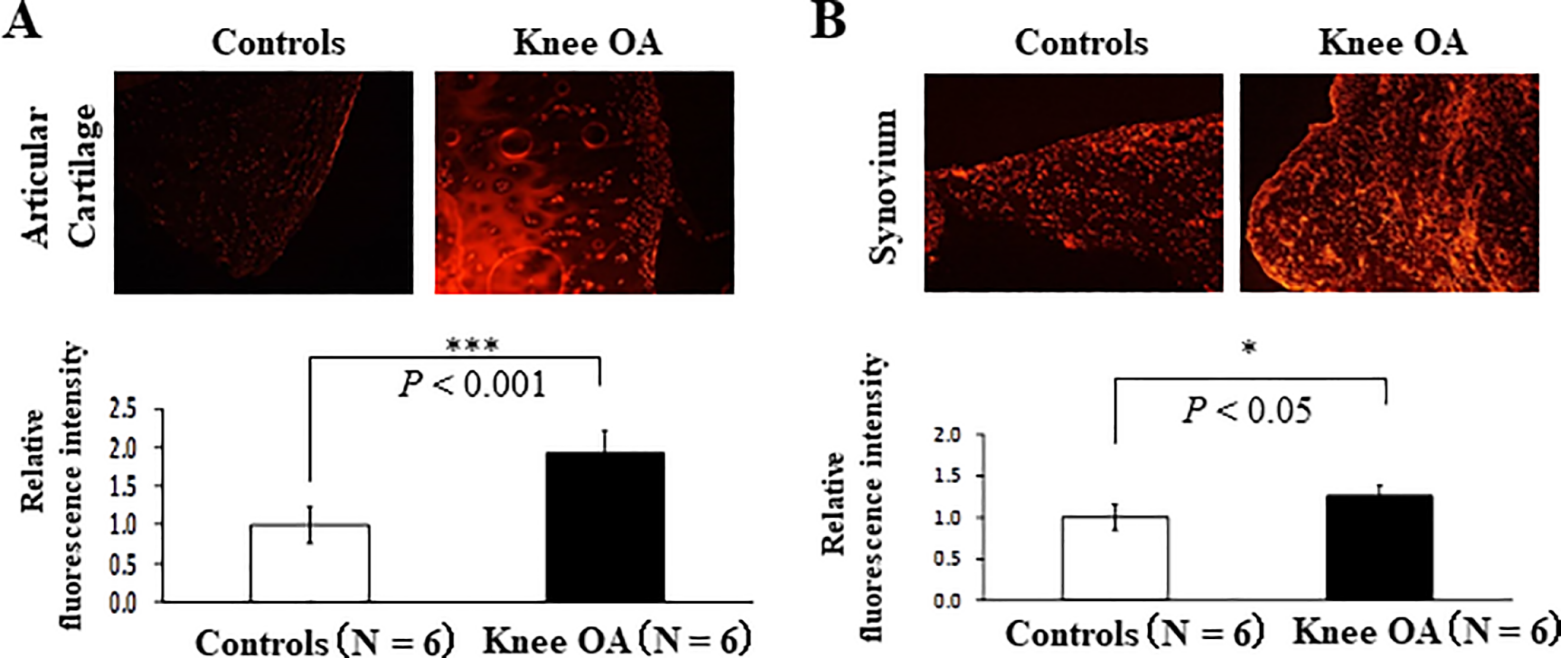
Intracellular reactive oxygen species (ROS) generation in the cartilage and synovium of patients with end-stage knee osteoarthritis (OA). (A) Intracellular ROS generation in articular cartilage. Left and right panels show the findings of dihydroethidium (DHE) staining. Controls: anterior cruciate ligament reconstruction (ACLR) or medial meniscectomy (MM), Knee OA: total knee arthroplasty (TKA) (N = 6, ***P < 0.001 versus controls, Student’s t-test). Error bars show the mean ± standard deviation. (B) Intracellular ROS generation in synovium. Left and right panels show the findings of DHE staining. Controls: ACLR or MM, Knee OA: TKA (N = 6, *P < 0.05 versus controls, Student’s t-test). Error bars show the mean ± standard deviation.

The intensity of DHE staining in the knee cartilage affected by end-stage knee OA was 1.94-fold higher than that in control cartilage (Fig 1A and Table 1). The intensity of DHE staining in the synovium affected by end-stage knee OA was 1.26-fold higher than that in control synovium (Fig 1B and Table 1). These results indicate that intracellular ROS generation significantly increased in the whole knee joint.

**Table 1:** Baseline characteristics of patients from Fig 1.

Characteristics	Controls (N = 6)	Knee OA(N = 6)	P value
**Age (years)**	31.6±13.2	72.6±7.03	< 0.01
**Gender (Male/female)**	Male 4, Female 2	Male 0, Female 6	
**BMI (kg/m**^**2**^**)**	23.3±2.0	25.4±3.9	0.26

OA: osteoarthritis, BMI: body mass index

### Significantly lower SOD activity in knee cartilage and synovium of patients with OA than in control cartilage and synovium

**Fig 2:**
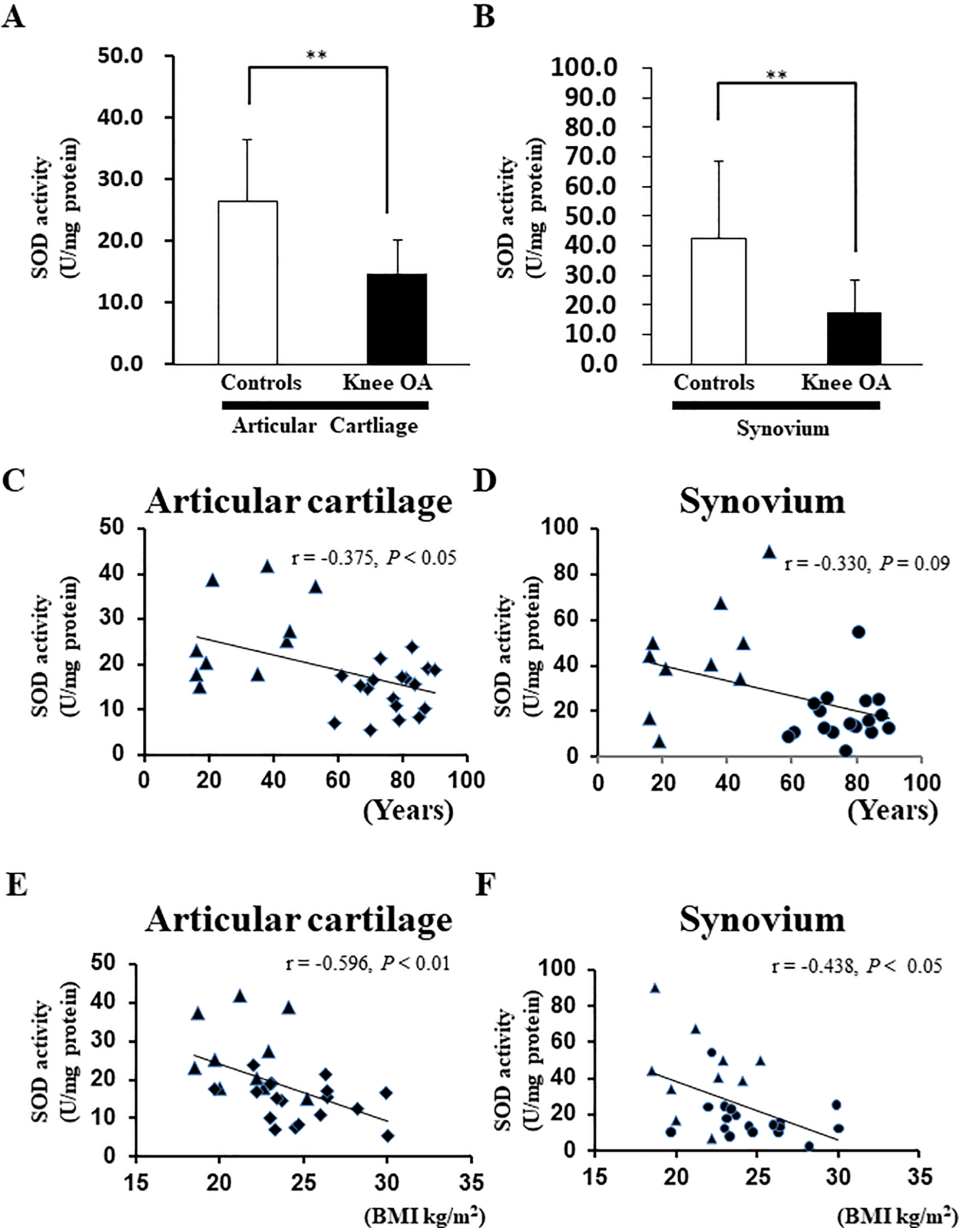
Superoxide dismutase (SOD) activity in the cartilage and synovium of patients with end-stage knee osteoarthritis (OA). (A) SOD activity in articular cartilage. Controls: anterior cruciate ligament reconstruction (ACLR), Knee OA: total knee arthroplasty (TKA) (***P < 0.001 versus controls, Mann–Whitney U test). Error bars show the mean ± standard deviation. (B) SOD activity in synovium. Controls: ACLR, Knee OA: TKA (N = 6, **P < 0.01 versus controls, Mann–Whitney U test). Error bars show the mean ± standard deviation. (C) Correlation between the SOD activity in cartilage and age. (▲) indicates cartilage from patients who underwent ACLR. (◆) indicates cartilage from patients who underwent TKA. Spearman’s correlation method was used to assess the correlation between the SOD activity in cartilage and age. (D) Correlation between the SOD activity in synovium and age. (▲) indicates synovium from patients who underwent ACLR. (●) indicates synovium from patients who underwent TKA. Spearman’s correlation method was used to assess the correlation between the SOD activity and age. (E) Correlation between the SOD activity and body mass index (BMI) in cartilage. (▲) indicates cartilage from patients who underwent ACLR. (◆) indicates cartilage from patients who underwent TKA. (F) Correlation between the SOD activity in synovium and BMI. (▲) indicates synovium from patients who underwent ACLR. (●) indicates synovium from patients who underwent TKA. Spearman’s correlation method was used to assess the correlation between the SOD activity and age.

We also evaluated the SOD activity in the articular cartilage and synovium of patients with knee OA. The SOD activity in the knee articular cartilage was significantly lower than that in the control group (Fig 2A). Furthermore, the SOD activity in the synovium was significantly lower than that in the controls (Fig 2B). The mean age of the patients who underwent TKA was significantly higher than that of the patients who underwent ACLR (Table 2). Therefore, we cannot deny the possibility that aging induced a decrease in the SOD activity level in the cartilage and synovium of patients with end-stage OA. Spearman’s correlation method was used to clarify the correlation between age and SOD activity. The SOD activity in the cartilage showed a weak negative correlation with age (r = −0.375, *P* < 0.05) (Fig 2C). However, there was no significant correlation between the SOD activity in the synovium and age (r = −0.330, *P* = 0.09) (Fig 2D). Meanwhile, the SOD activity in the knee cartilage showed a moderate negative correlation with BMI (r = −0.596, *P* < 0.01) (Fig 2E). Furthermore, the SOD activity in the knee synovium showed a moderate negative correlation with BMI (r = −0.438, *P* < 0.05) (Fig 2F).

**Table 2:** Baseline characteristics of patients from Fig 2.

Characteristics	Controls (N = 10)	Knee OA (N = 18)	P value
**Age (years)**	30.4±14.1	76.8±9.1	< 0.001
**Gender (Male/female)**	Male 5, Female 5	Male 0, Female 18	
**BMI (kg/m**^**2**^**)**	21.5±2.3	24.8±2.8	< 0.01

OA: osteoarthritis, BMI: body mass index

### Significantly lower SOD activity in the femoral head cartilage of patients with hip OA than femoral head fractures, independent of age

**Fig 3:**
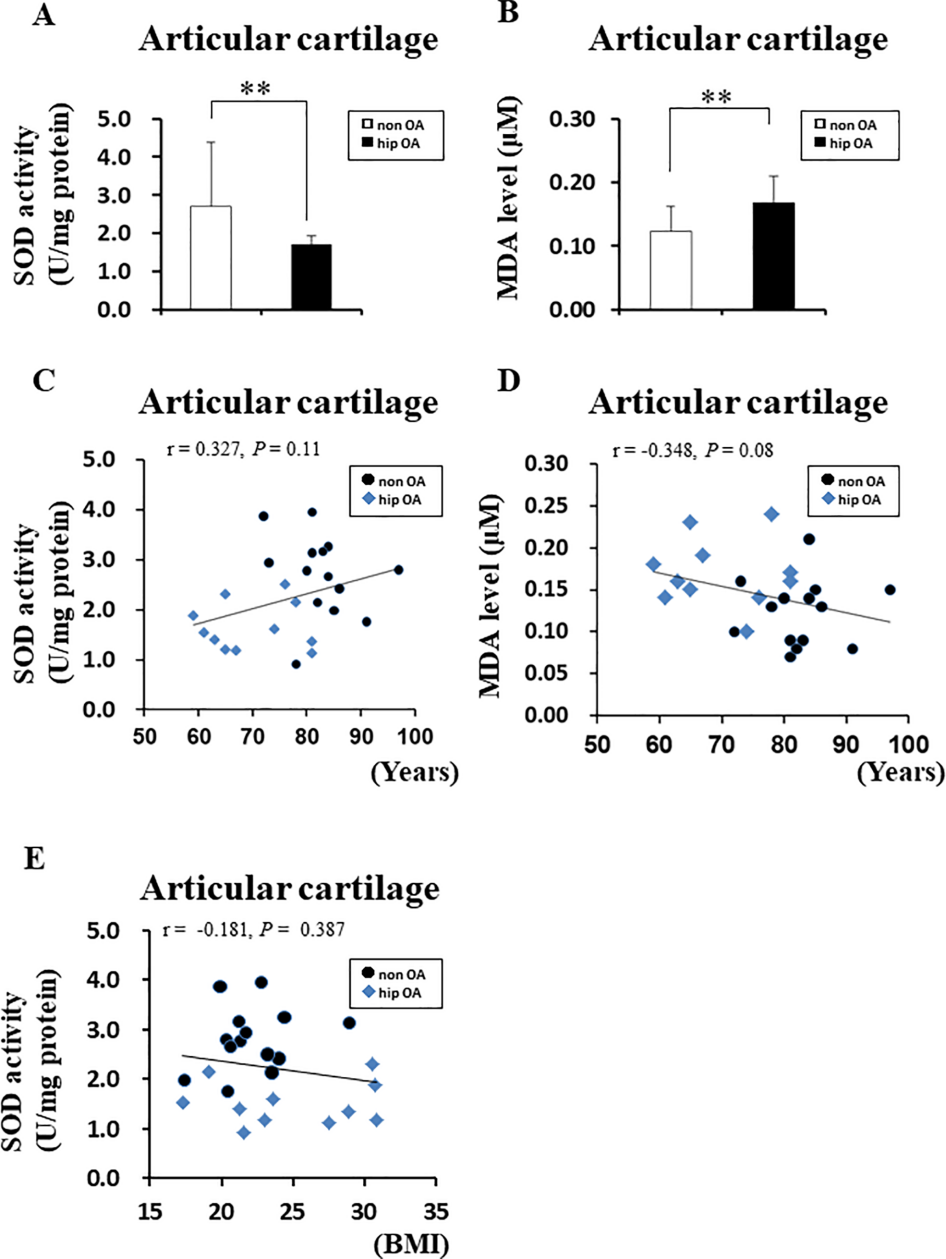
Superoxide dismutase (SOD) activity and malondialdehyde (MDA) level in the cartilage of patients with end-stage hip OA. (A) Comparison of SOD activity in cartilage from patients with femoral neck fractures (non-OA) and patients with hip OA. non-OA: cartilage from patients with femoral neck fractures, hip OA: cartilage from patients with end-stage hip OA (**P < 0.01 versus controls, Mann–Whitney U test). Error bars show the mean ± standard deviation. (B) Comparison of MDA level in cartilage from patients with non-OA and hip OA (**P < 0.01 versus controls, Student’s t-test). Error bars show the mean ± standard deviation. (C) Correlation between SOD activity in cartilage and age. Spearman’s correlation method was used to assess the correlation between SOD activity in cartilage and age. (D) Correlation between the MDA level in cartilage and age. Spearman’s correlation method was used to assess the correlation between the MDA level in cartilage and age. (E) Correlation between SOD activity in cartilage and body mass index (BMI). Spearman’s correlation method was used to assess the correlation between SOD activity in cartilage and BMI.

To investigate the correlation between SOD activity and aging, we focused on the femoral head cartilage, for which it is easy to eliminate the effects of aging. We aimed to prove the causal relationship of SOD activity with aging by comparing the SOD activity in the femoral head cartilage of patients without OA who had a femoral neck fracture and the SOD activity in the femoral head cartilage of patients with hip OA. The average age of patients with femoral neck fractures was significantly higher than that of patients with end-stage hip OA (Table 3). However, the SOD activity in the femoral head cartilage of patients with hip OA was significantly lower than that of patients with femoral head fractures (Fig 3A). Furthermore, the MDA level, which is an oxidized lipid marker, was significantly higher in the cartilage of patients with than without hip OA (Fig 3B). Next, the relationship between aging and SOD activity in the cartilage and the relationship between aging and MDA in the cartilage were evaluated. We found no significant correlation between age and SOD activity in the cartilage (r = 0.327, *P* = 0.11) (Fig 3C). Moreover, there was no significant correlation between age and the MDA level (r = −0.348, *P* = 0.08) (Fig 3D). Furthermore, the relationship between SOD activity in the cartilage and BMI was evaluated. There was no significant correlation between the SOD activity in the femoral head cartilage and BMI (r = −0.181, *P* = 0.387) (Fig 3E). These data suggest that the SOD activity of the hip articular cartilage decreases independent of age or BMI.

**Table 3:** Baseline characteristics of patients from Fig 3.

Characteristics	non OA (N = 14)	hip OA (N = 10)	P value
**Age (years)**	82.64±6.42	70.0±8.17	< 0.001
**Gender (Male/female)**	Male 3, Female 11	Male 0, Female 11	
**BMI (kg/m**^**2**^**)**	22.0±2.69	25.0±4.85	0.06

OA: osteoarthritis, BMI: body mass index

## Discussion

OA is the most prominent joint disorder that increases with aging. Aging, obesity, mechanical stress, smoking, and a postmenopausal status in women are well-known risk factors for OA. These risk factors have been found to induce ROS production [11–14]. Recent studies have concluded that ROS-inducible oxidative stress causes subchondral bone dysfunction, intracellular signaling, synovial tissue inflammation, low matrix synthesis, cartilage degeneration, mitochondrial dysfunction, apoptosis, and chondrocyte cellular senescence [15]. In fact, some researchers have reported that oxidative stress is significantly increased in OA-affected cartilage [16–18]. We previously reported that ROS generated from chondrocytes themselves induce chondrocyte dysfunction, resulting in cartilage degeneration [9]. In the present study, we found that intracellular ROS generation in the cartilage of patients with end-stage knee OA was significantly increased. In this study, we evaluated oxidative stress in knee OA and hip OA cartilage using DHE staining and MDA. DHE is generally known to specifically dye superoxide. However, a recent report indicated that accurate quantities of superoxide cannot be quantified unless the superoxide is stained with DHE and evaluated by high-performance liquid chromatography [19]. MDA is one of many low-molecular-weight end-products of lipid hydroperoxide decomposition and is most often measured as an oxidative stress biomarker [20]. However, MDA is not a specific oxidative stress marker [21], so identification of the exact type of ROS is a future task. In any case, the intracellular ROS increased in the end-stage knee OA and the hip OA, respectively, and the oxidative stress was increased (Figs 1 and 3B).

The cellular elements of the synovium are a major source of joint fluid components. Cartilage degeneration is considered to be the main pathogenetic source of OA. However, how oxidative stress in the synovium affects OA progression has not been determined. In the present study, we revealed that intracellular ROS generation was significantly higher in the synovium of patients with than without OA (Fig 1B). This result suggests the possibility that OA-affected synovium strongly affects OA progression. Although we consider that intracellular ROS are the most significant cause of cartilage degeneration, intracellular ROS generation from synoviocytes also strongly contributes to cartilage degeneration.

Both the hip joint and knee joint are weight-bearing joints. BMI is a major risk factor for OA [22]. Another group reported a positive association between an increased BMI and the risk of hip OA, and a weaker positive association was observed for hip OA than knee OA [23]. In the present study, the BMI in patients with knee OA was significantly higher than that in the control group (Table 2). Additionally, we found no significant difference in the BMI between patients with hip OA and patients without OA (Table 3). These data support the findings in recent reports. Examination of the relationship between BMI and SOD activity reveals that the SOD activity in the knee cartilage showed a moderate negative correlation with BMI (Fig 2E). Furthermore, the SOD activity in the knee synovium showed a moderate negative correlation with BMI (Fig 2F). Conversely, no significant correlation was found between the SOD activity in the femoral head cartilage and BMI (Fig 3E). These results suggest that the SOD activity in cartilage and synovium significantly decreased in OA independent of the BMI.

We found that the MDA level was significantly higher in the cartilage of patients with hip OA than in the cartilage of patients with femoral neck fractures but without hip OA (Fig 3B). Regan *et al*. reported that SOD3 was significantly lower at the protein level in the articular cartilage of patients with than without hip OA [17]. We found that SOD activity was significantly lower in the knee cartilage and synovium of patients with knee OA as well as in the hip cartilage of patients with hip OA (Figs 2A, 2B and 3A). These data are consistent with those reported by Scott *et al*. [6], who found that SOD1, SOD2, and SOD3 were decreased in OA-affected knee cartilage. These findings suggest that oxidative stress is high in the articular cartilage of weight-bearing joints regardless of whether the knee joint or hip joint is affected.

Proteins are synthesized by translation. A study performed in 2010 showed that diversity is created in the function and structure of the protein by various post-translational modifications that occur after the protein is synthesized [24]. SOD has three isozymes, and when SOD1, SOD2, and SOD3 are subjected to various post-translational modifications such as nitration, glycation, glutathionylation, and phosphorylation, the SOD activity reportedly decreases [24]. Fu *et al*. recently reported that in a rat model, SOD2 activity declined owing to increased acetylation of lysine, which is a post-translational modification, despite an increased amount of SOD2 protein in cartilage with aging [25]. Based on these findings, we focused on assessing activity rather than gene expression and protein amount.

Aging is the largest risk factor for OA [26]. In this study, the average age was significantly higher in the knee OA group than in the control group (Tables 1 and 2). One report indicated that SOD activity decreases with age [27]. Even if the SOD activity in the cartilage and synovium in the OA group was significantly low, it was difficult to judge whether the SOD activity was low owing to aging or OA because the average age of patients in the knee OA group was high. However, in the analysis of the hip joint cartilage, the average age of the patients in the hip OA group was significantly lower than that of patients with femoral neck fractures used as controls (Table 3). Nevertheless, because SOD activity was significantly lower in the hip OA group (Fig 3A), SOD activity was proven to be decreased in OA cartilage independent of age-related change.

The SOD activity measurement method using the Cayman Chamicals assay has a relatively narrow detection range, but it is excellent with respect to dilution linearity of the specimen. Furthermore, we also conducted a SOD addition recovery test (tests of specimens spiked with exogenous SOD). Following addition of SOD, the measured SOD values rose in a concentration-dependent manner (S3A Fig). Absorbance inhibition was also seen in a highly reproducible concentration-dependent manner (S3B Fig). The measurement of SOD activity using the NWLSS assay is based on the auto-oxidation of hematoxylin, and so there is no calibration curve. SOD activity is calculated from the rate of inhibition of auto-oxidation at the time of sample addition. In this study, we performed measurements using both the NWLSS assay (the hematoxylin auto-oxidation method) and the Cayman Chemicals assay (a more sensitive tetrazolium method), and we found a positive correlation between the two (S4 Fig). For SOD activity in hip joint cartilage, the more sensitive Cayman Chemicals kit was used (Fig 3). In order to make SOD activity a meaningful biomarker, it is necessary to find a clearly threshold that can distinguish between OA and non-OA. It would be difficult to determine a threshold that can separate OA from non-OA unless SOD activity for each stage of OA (early, advanced and end-stage OA) was evaluated. Since patients who undergo surgery are mostly in the end stage of OA and patients with early or advanced stage OA rarely undergo surgery, it is difficult to acquire early and advanced stage tissue samples. Evaluating SOD activity for each stage of OA in order to find thresholds that can distinguish between OA and non-OA is a future task. Furthermore, if the SOD activity in the joints and whole body can be clarified by evaluating SOD activity in serum, joint fluid, articular cartilage, and synovium, then SOD can be expected to serve as a biomarker of OA. Our results to date showing that the total SOD activity is decreased in end-stage OA are unequivocal.

## Supporting information

S1 Fig This is a photograph taken during the performance of artificial knee arthroplasty. Cartilage and synovium were collected from the outside of the patellofemoral joint. Articular cartilage and synovium of a patient who underwent medial meniscectomy or anterior cruciate ligament reconstruction (used as the control group) were collected from the same site.
(TIF)

S2 Fig This photograph shows the removed femoral head. A piece of cartilage was scraped from the superficial layer of the femoral head and collected.
(TIF)

S3 Fig SOD addition recovery test (specimens spiked with exogenous SOD). 5 μL of a SOD preparation was added to 50 μL of a 20-fold dilution of cartilage disruption supernatant, and assayed with the SOD assay kit (Cayman Chemicals kit). The data confirm that, following addition of exogenous SOD, the measured SOD value rises in a concentration-dependent manner (A). Absorbance inhibition also occurs in a concentration-dependent manner (B).
(TIF)

S4 Fig SOD activity tested by two different assays. S4 Fig shows the correlation between data obtained using the two SOD kits to assay femoral head cartilage. There was a high positive correlation between SOD activity measured with the Cayman Chemicals kit and with the NWLSS kit (r = 0.828, p < 0.001, n = 17).
(TIF)

S1 Table Individual data of samples stained with dihydroethidium (DHE). Nos. 1 to 6 are defined as the non-OA groups. Nos. 7 to 12 are defined as the knee OA groups.
(DOC)

S2 Table Individual data of patients with knee osteoarthritis (OA) who underwent total knee arthroplasty (TKA) and patients who underwent anterior cruciate ligament (ACL) reconstruction. Nos. 1 to 10 are defined as the non-OA groups. Nos. 11 to 28 are defined as the knee OA groups.
(DOC)

S3 Table Individual data of patients with hip osteoarthritis (OA) who underwent total hip arthroplasty (THA) and patients with femoral neck fractures who underwent bipolar hip arthroplasty. Nos. 1 to 14 are defined as the non-OA groups. Nos. 15 to 25 are defined as the hip OA groups.
(DOC)
