# Supplementary figures and images for: Superoxide dismutase activity is significantly lower in end-stage osteoarthritic cartilage than non-osteoarthritic cartilage
Source: PLoS One. 2018 Sep 17;13(9):e0203944. doi: 10.1371/journal.pone.0203944 (PMC6141073; doi:10.1371/journal.pone.0203944)

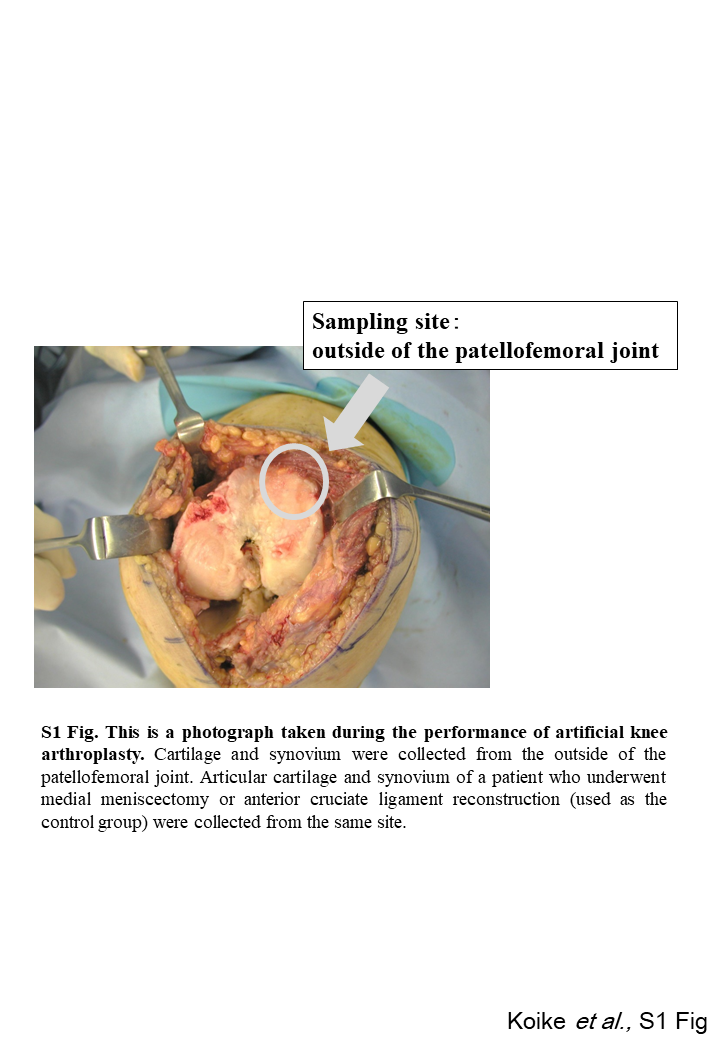

Supplement: S1 Fig — Cartilage and synovium were collected from the outside of the patellofemoral joint. Articular cartilage and synovium of a patient who underwent medial meniscectomy or anterior cruciate ligament reconstruction (used as the control group) were collected from the same site. (TIF) [file pone.0203944.s001.TIF]

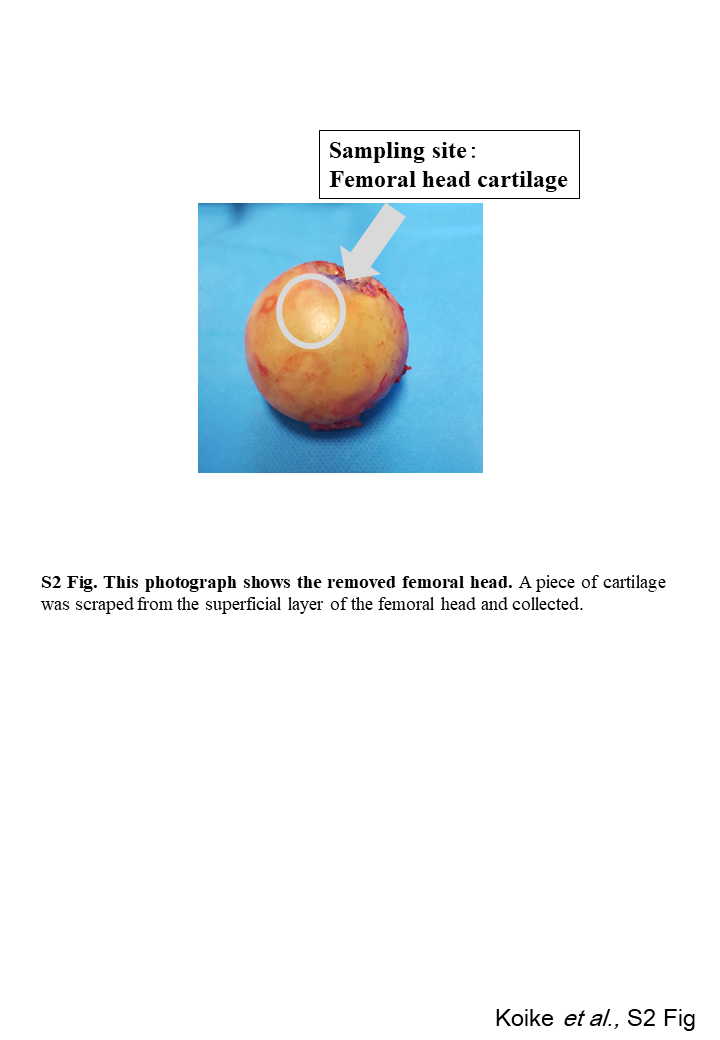

Supplement: S2 Fig — A piece of cartilage was scraped from the superficial layer of the femoral head and collected. (TIF) [file pone.0203944.s002.TIF]

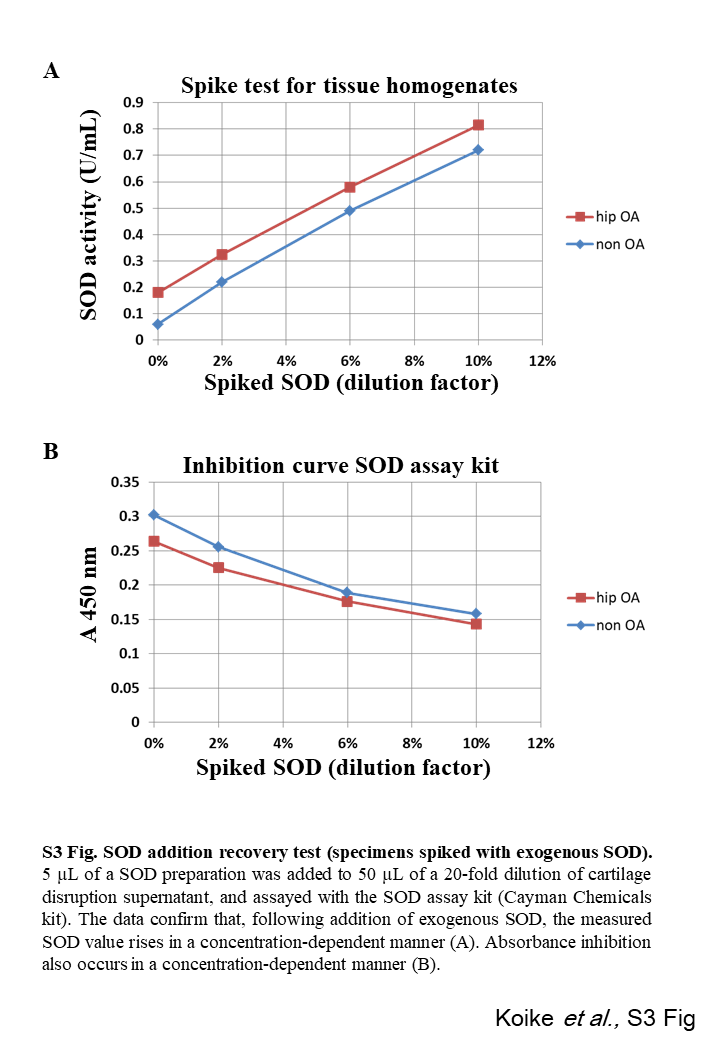

Supplement: S3 Fig — 5 μL of a SOD preparation was added to 50 μL of a 20-fold dilution of cartilage disruption supernatant, and assayed with the SOD assay kit (Cayman Chemicals kit). The data confirm that, following addition of exogenous SOD, the measured SOD value rises in a concentration-dependent manner (A). Absorbance inhibition also occurs in a concentration-dependent manner (B). (TIF) [file pone.0203944.s003.TIF]

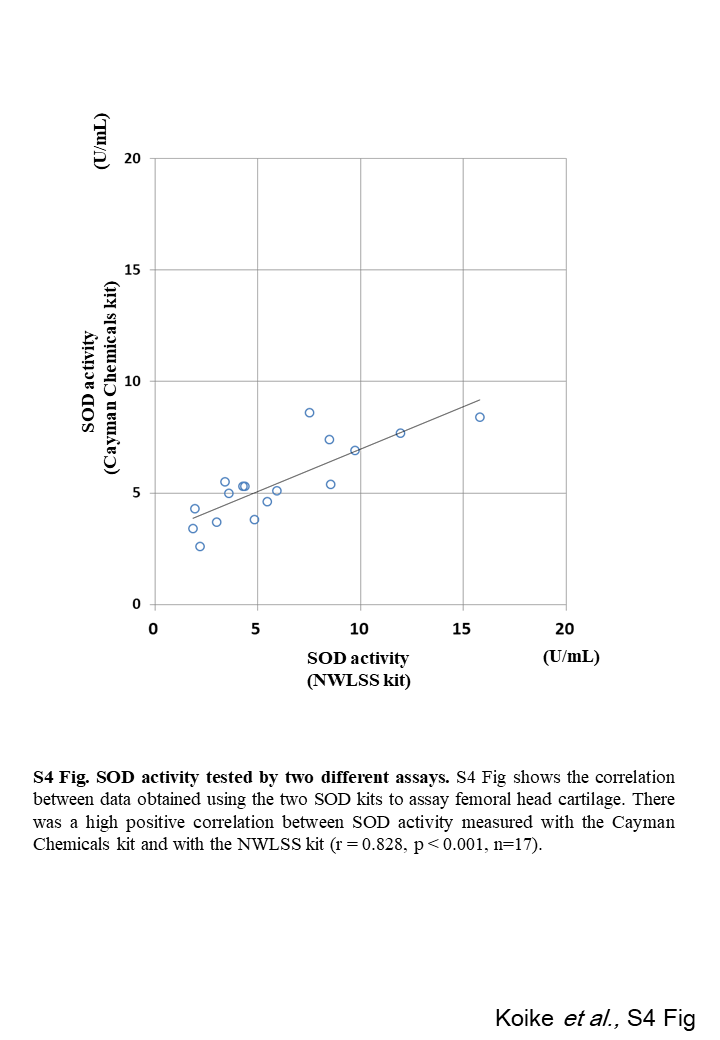

Supplement: S4 Fig — shows the correlation between data obtained using the two SOD kits to assay femoral head cartilage. There was a high positive correlation between SOD activity measured with the Cayman Chemicals kit and with the NWLSS kit (r = 0.828, p < 0.001, n = 17). (TIF) [file pone.0203944.s004.TIF]
