# Supplementary material for: Superoxide dismutase activity is significantly lower in end-stage osteoarthritic cartilage than non-osteoarthritic cartilage
Source: PLoS One. 2018 Sep 17;13(9):e0203944. doi: 10.1371/journal.pone.0203944 (PMC6141073; doi:10.1371/journal.pone.0203944)
Supplement: S1 Table — Nos. 1 to 6 are defined as the non-OA groups. Nos. 7 to 12 are defined as the knee OA groups. (DOC) [file pone.0203944.s005.doc]

**S1 Table. Individual data of samples stained with dihydroethidium (DHE).**

| **No.** | **Age**  **(years)** | **Gender** | **Operation** | **Kellgren**  **And**  **Lawrence grade (KL)** | **Height**  **(cm)** | **Body weight (kg)** | **BMI**  **(kg/m^2^)** |
| --- | --- | --- | --- | --- | --- | --- | --- |
| **1** | 18 | M | ACL reconstruction | 0 | 178 | 68 | 21.5 |
| **2** | 35 | M | ACL reconstruction | 1 | 180 | 80 | 24.7 |
| **3** | 21 | M | Meniscectomy | 0 | 178 | 75.3 | 23.8 |
| **4** | 42 | F | Meniscectomy | 1 | 163 | 57 | 21.5 |
| **5** | 42 | M | ACL reconstruction | 0 | 173 | 79 | 26.4 |
| **6** | 52 | F | ACL reconstruction | 1 | 157 | 54 | 21.9 |
| **7** | 69 | F | TKA | 4 | 148 | 65.4 | 29.9 |
| **8** | 76 | F | TKA | 4 | 154 | 65.4 | 27.6 |
| **9** | 66 | F | TKA | 4 | 150 | 42 | 18.7 |
| **10** | 84 | F | TKA | 4 | 143.3 | 55.6 | 27.1 |
| **11** | 66 | F | TKA | 4 | 150 | 58 | 25.8 |
| **12** | 75 | F | TKA | 4 | 146 | 49.8 | 23.4 |

Nos. 1 to 6 are defined as the non-OA groups. Nos. 7 to 12 are defined as the knee OA groups.
