# Supplementary material for: Superoxide dismutase activity is significantly lower in end-stage osteoarthritic cartilage than non-osteoarthritic cartilage
Source: PLoS One. 2018 Sep 17;13(9):e0203944. doi: 10.1371/journal.pone.0203944 (PMC6141073; doi:10.1371/journal.pone.0203944)
Supplement: S2 Table — Nos. 1 to 10 are defined as the non-OA groups. Nos. 11 to 28 are defined as the knee OA groups. (DOC) [file pone.0203944.s006.doc]

**S2 Table. Individual data of patients with knee osteoarthritis (OA) who underwent total knee arthroplasty (TKA) and patients who underwent anterior cruciate ligament (ACL) reconstruction.**

| **No.** | **Age** | **Gender** | **Operation** | **Kellgren**  **and**  **Lawrence grade**  **(KL)** | **Height**  **(cm)** | **body weight**  **(kg)** | **BMI**  **(kg/m^2^)** | **SOD activity in cartilage**  **(U/mg protein)** | **SOD activity**  **in synovium**  **(U/mg protein)** |
| --- | --- | --- | --- | --- | --- | --- | --- | --- | --- |
| **1** | 35 | M | ACL reconstruction | 0 | 176 | 70 | 22.6 | 17.9 | 40.6 |
| **2** | 19 | F | ACL reconstruction | 0 | 156 | 54 | 22.2 | 20.4 | 7.0 |
| **3** | 17 | M | ACL reconstruction | 0 | 169 | 72 | 25.2 | 15.1 | 50.0 |
| **4** | 16 | M | ACL reconstruction | 0 | 174.5 | 61 | 20.0 | 17.8 | 17.0 |
| **5** | 44 | M | ACL reconstruction | 0 | 161 | 51 | 19.7 | 25.2 | 34.2 |
| **6** | 53 | F | ACL reconstruction | 0 | 148 | 41 | 18.7 | 37.3 | 90.0 |
| **7** | 38 | M | ACL reconstruction | 0 | 171 | 62 | 21.2 | 41.8 | 67.5 |
| **8** | 21 | F | ACL reconstruction | 0 | 173 | 72 | 24.1 | 38.8 | 38.7 |
| **9** | 16 | F | ACL reconstruction | 0 | 161 | 47.9 | 18.5 | 23.1 | 44.3 |
| **10** | 45 | F | ACL reconstruction | 1 | 175 | 70 | 22.9 | 27.4 | 50.0 |
| **11** | 61 | F | TKA | 4 | 161 | 51 | 19.7 | 17.6 | 10.8 |
| **12** | 87 | F | TKA | 3 | 149 | 51 | 23.0 | 10.1 | 25.0 |
| **13** | 73 | F | TKA | 4 | 142 | 53.1 | 26.3 | 21.4 | 10.4 |
| **14** | 79 | F | TKA | 3 | 154 | 58 | 24.5 | 7.6 | 13.5 |
| **15** | 59 | F | TKA | 4 | 148 | 51 | 23.3 | 7.1 | 8.4 |
| **16** | 88 | F | TKA | 3 | 147 | 50 | 23.1 | 19.1 | 18.0 |
| **17** | 81 | F | TKA | 4 | 144 | 46 | 22.2 | 16.9 | 54.4 |
| **18** | 83 | F | TKA | 3 | 140.7 | 43.5 | 22.0 | 23.8 | 24.5 |
| **19** | 85 | F | TKA | 4 | 138 | 47 | 24.7 | 8.4 | 10.5 |
| **20** | 77 | F | TKA | 4 | 147 | 60.95 | 28.2 | 12.5 | 2.5 |
| **21** | 69 | F | TKA | 4 | 157 | 58.3 | 23.7 | 14.6 | 19.7 |
| **22** | 84 | F | TKA | 4 | 147 | 57.1 | 26.4 | 15.5 | 15.8 |
| **23** | 80 | F | TKA | 4 | 147 | 57 | 26.4 | 17.1 | 13.3 |
| **24** | 70 | F | TKA | 3 | 158 | 75 | 30.0 | 5.5 | 12.4 |
| **25** | 78 | F | TKA | 4 | 152 | 60 | 26.0 | 10.9 | 14.3 |
| **26** | 67 | F | TKA | 4 | 156 | 57 | 23.4 | 15.2 | 23.4 |
| **27** | 71 | F | TKA | 4 | 147.5 | 65 | 29.9 | 16.6 | 25.4 |
| **28** | 90 | F | TKA | 4 | 140 | 45 | 23.0 | 18.9 | 12.6 |

Nos. 1 to 10 are defined as the non-OA groups. Nos. 11 to 28 are defined as the knee OA groups.
