# Supplementary material for: Superoxide dismutase activity is significantly lower in end-stage osteoarthritic cartilage than non-osteoarthritic cartilage
Source: PLoS One. 2018 Sep 17;13(9):e0203944. doi: 10.1371/journal.pone.0203944 (PMC6141073; doi:10.1371/journal.pone.0203944)
Supplement: S3 Table — Nos. 1 to 14 are defined as the non-OA groups. Nos. 15 to 25 are defined as the hip OA groups. (DOC) [file pone.0203944.s007.doc]

**S3 Table. Individual data of patients with hip osteoarthritis (OA) who underwent total hip arthroplasty (THA) and patients with femoral neck fractures who underwent bipolar hip arthroplasty.**

| **No.** | **Age**  **(years)** | **Gender** | **Operation** | **Kellgren**  **and**  **Lawrence grade**  **(KL)** | **Height**  **(cm)** | **Body weight**  **(kg)** | **BMI**  **(kg/m^2^)** | **SOD activity (U/mg protein)** | **MDA (μM)** |
| --- | --- | --- | --- | --- | --- | --- | --- | --- | --- |
| **1** | 85 | F | BHA | 1 | 151 | 39.7 | 17.4 | 1.98 | 0.15 |
| **2** | 84 | F | BHA | 1 | 156.3 | 59.6 | 24.4 | 3.26 | 0.14 |
| **3** | 72 | F | BHA | 1 | 145 | 41.8 | 19.9 | 3.87 | 0.1 |
| **4** | 78 | F | BHA | 1 | 155 | 52 | 21.6 | 0.91 | 0.13 |
| **5** | 81 | M | BHA | 1 | 165 | 62 | 22.8 | 3.95 | 0.07 |
| **6** | 97 | F | BHA | 1 | 142 | 41 | 20.3 | 2.8 | 0.15 |
| **7** | 81 | F | BHA | 1 | 140 | 56.65 | 28.9 | 3.14 | 0.09 |
| **8** | 83 | F | BHA | 1 | 151 | 48.4 | 21.2 | 3.17 | 0.09 |
| **9** | 86 | F | BHA | 1 | 152 | 55.5 | 24.0 | 2.42 | 0.13 |
| **10** | 82 | F | BHA | 1 | 160 | 60.1 | 23.5 | 2.14 | 0.08 |
| **11** | 91 | M | BHA | 1 | 163 | 54.2 | 20.4 | 1.76 | 0.08 |
| **12** | 80 | M | BHA | 1 | 151.5 | 48.86 | 21.3 | 2.78 | 0.14 |
| **13** | 73 | F | BHA | 1 | 151 | 49.4 | 21.7 | 2.94 | 0.16 |
| **14** | 84 | F | BHA | 1 | 158 | 51.4 | 20.6 | 2.66 | 0.21 |
| **15** | 59 | F | THA | 4 | 152 | 71 | 30.7 | 1.88 | 0.18 |
| **16** | 78 | F | THA | 4 | 152.7 | 44.5 | 19.1 | 2.15 | 0.24 |
| **17** | 65 | F | THA | 4 | 163 | 81.8 | 30.8 | 1.19 | 0.15 |
| **18** | 81 | F | THA | 4 | 140.6 | 54.4 | 27.5 | 1.13 | 0.17 |
| **19** | 63 | F | THA | 4 | 168 | 60 | 21.3 | 1.39 | 0.16 |
| **20** | 81 | F | THA | 4 | 144.5 | 60.4 | 28.9 | 1.36 | 0.16 |
| **21** | 65 | F | THA | 4 | 156.8 | 75 | 30.5 | 2.3 | 0.23 |
| **22** | 67 | F | THA | 4 | 149.2 | 51.2 | 23.0 | 1.18 | 0.19 |
| **23** | 76 | F | THA | 4 | 155 | 55.8 | 23.2 | 2.5 | 0.14 |
| **24** | 61 | F | THA | 4 | 155 | 41.6 | 17.3 | 1.53 | 0.14 |
| **25** | 74 | F | THA | 4 | 149 | 52.5 | 23.6 | 1.6 | 0.1 |

Nos. 1 to 14 are defined as the non-OA groups. Nos. 15 to 25 are defined as the hip OA groups.
